# Supplementary material for: Identification of photoperiod-induced specific miRNAs in the adrenal glands of Sunite sheep (Ovis aries)
Source: Front Vet Sci. 2022 Jul 22;9:888207. doi: 10.3389/fvets.2022.888207 (PMC9354845; doi:10.3389/fvets.2022.888207)
Supplement: Supplementary file 1 [file Data_Sheet_1.ZIP › Supplementary file/miRDeep2 file.zip!/miRDeep2 file/Novel_Structure/SP42A1/Novel_574.pdf]

Provisional ID	:	NC_019459.2_3359
Score total	:	1.9
Score for star read(s)	:	-1.3
Score for read counts	:	0
Score for mfe	:	1.6
Score for randfold	:	1.6
Score for cons. seed	:	
Total read count	:	63
Mature read count	:	63
Loop read count	:	0
Star read count	:	0

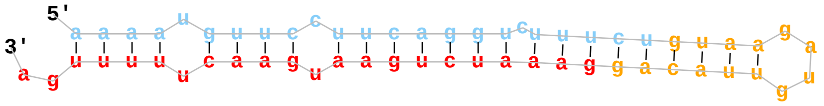

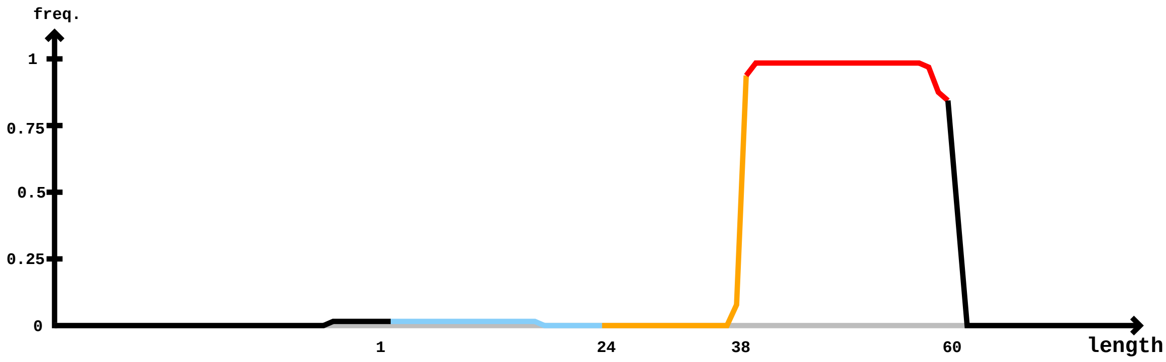


```
                                Star                              Mature
5'- cugggaagcccucauacugcuauuggguuggccaaaauguuccuucaggucuuucuguaagauguuacaggaaaucugaaugaacuuuuugaugaauccaguguuaaucau   -3'   exp
    ..(((...)))..........(((((((((.(.(((((.((((.(((((((.(((((((((....)))))))))))))))).)))).))))).).)))))))))........   reads   mm   sample
    ...........................uuggccaaaauguucGuucagg..............................................................   1       1    seq
    .....................................................................ggaaaCcugaaugaacuuuu......................   1       1    seq
    .....................................................................ggaaaCcugaaugaacuuuuu.....................   4       1    seq
    ......................................................................gaaaCcugaaugaacuuuuug....................   2       1    seq
    ......................................................................Aaaaucugaaugaacuuuuuga...................   52      1    seq
    ......................................................................gaaaucugaaugaacuuuuuga...................   1       0    seq
    .......................................................................aaaucugaaugaacuuuuu.....................   1       0    seq
    .......................................................................aaaucuAaaugaacuuuuu.....................   1       1    seq
    .......................................................................aaaCcugaaugaacuuuuuga...................   1       1    seq
```
